# Single Halide Perovskite/Semiconductor Core/Shell Quantum Dots with Ultrastability and Nonblinking Properties

**DOI:** 10.1002/advs.201900412

**Published:** 2019-07-01

**Authors:** Xiaosheng Tang, Jie Yang, Shiqi Li, Zhengzheng Liu, Zhiping Hu, Jiongyue Hao, Juan Du, Yuxin Leng, Haiyan Qin, Xing Lin, Yue Lin, Yuxi Tian, Miao Zhou, Qihua Xiong

**Affiliations:** ^1^ Key Laboratory of Optoelectronic Technology and Systems (Ministry of Education) College of Optoelectronic Engineering Chongqing University Chongqing 400044 China; ^2^ State Key Laboratory of High Field Laser Physics Shanghai Institute of Optics and Fine Mechanics Chinese Academy of Sciences Shanghai 201800 China; ^3^ Center of Materials Science and Optoelectronics Engineering University of Chinese Academy of Sciences Beijing 100049 China; ^4^ Center for Chemistry of Nover and High‐Performance Materials and Department of Chemistry Zhejiang University Hangzhou 310027 P. R. China; ^5^ Hefei National Laboratory for Physical Sciences at the Microscale University of Science and Technology of China Hefei 230026 China; ^6^ School of Chemistry and Chemical Engineering Key Laboratory of Mesoscopic Chemistry of MOE and Jiangsu Key Laboratory of Vehicle Emissions Control Nanjing University Nanjing 210023 China; ^7^ Division of Physics and Applied Physics School of Physical and Mathematical Sciences Nanyang Technological University Singapore 637371 Singapore

**Keywords:** core/shell structure, first‐principles, halide perovskites, nonblinking, quantum dots

## Abstract

The further practical applications of halide perovskite quantum dots (QDs) are blocked by problems of instability and nonradiative Auger recombination manifested as photoluminescence blinking. Here, single core/shell structured perovskite semiconductor QDs are successfully fabricated by capping CsPbBr_3_ QD core with CdS shell. It is demonstrated that CsPbBr_3_/CdS core/shell QDs exhibit ultrahigh chemical stability and nonblinking photoluminescence with high quantum yield due to the reduced electronic traps within the core/shell structure. Efficiency of amplified spontaneous emission exhibits obvious enhancement compared to that of pure CsPbBr_3_ QDs, originating from the mitigated competition between stimulated emission and suppressed nonradiative biexciton Auger recombination. Furthermore, low‐threshold whispering‐gallery‐mode lasing with a high‐quality factor is achieved by incorporating CsPbBr_3_/CdS QDs into microtubule resonators. Density functional theory (DFT)‐based first‐principles calculations are also performed to reveal the atomic interface structure, which supports the existence of CsPbBr_3_/CdS structure. An interesting feature of spatially separated charge density at CsPbBr_3_/CdS interface is found, which may greatly contribute to the suppressed Auger recombination. The results provide a practical approach to improve the stability and suppress the blinking of halide perovskite QDs, which may pave the way for future applications for various optoelectronic devices.

Development of new functional materials becomes one major driving force for technology evolution. Recent years have witnessed a surge of research pertaining to organic–inorganic hybrid lead halide perovskites (CH_3_NH_2_PbX_3_, X = Cl, Br, I) and inorganic cesium lead halide perovskites (CsPbX_3_) as most attractive structures for optical gain materials in fields of photonics and optoelectronics.^1^, ^2^ Perovskite quantum dots (QDs) exhibit plenty of extraordinary electronic properties, including tunable bandgap, highly absorbance coefficient, and high photoluminescence quantum yield (PLQY),^3^ rendering great potential in light‐emitting diodes (LEDs),^4^ solar cells,^5^ photodetectors,^6^ and lasers.^7^ However, halide perovskites suffer from chemical instability due to their innate sensitivity to polar solvents and temperature,^8^, ^9^ and fluctuation of the fluorescence intensity due to the inner nonradiative Auger recombination process,^10^ which severely restrict their practical applications. In literature, great efforts have been made to increase the stability, such as by introducing mesoporous silica,^11^ intermolecular C=C bonding,^12^ alkyl phosphate layer,^13^ chemical doping,^14^ and heterostructures, such as CsPbBr_3_/ZnS heterodimers and CH_3_NH_2_PbX_3_/SiO_2_ sphere.^15^, ^16^

Also, these materials usually exhibit obvious photoluminescence (PL) blinking at the single‐dot level,^15^ which is caused by the nonradiative Auger recombination process^17^ and has severe detrimental effects on the efficiency of optoelectronic devices based on spontaneous emission (such as LEDs).^10^ In addition, competition between stimulated emission driven by biexciton recombination and nonradiative Auger recombination also limits the performance of devices based on stimulated emission, including micro/nanolasers.^17^ In this regard, suppressing nonradiative Auger recombination of perovskite QDs is critical to fulfill their potential for applications. Up to now, different approaches have been employed to different type of QDs, which include but are not limited to synthesizing dimer structures,^18^ adjusting content of oleic acid (OA)‐Cs precursor,^19^ and changing solution environment or surface ligand of QDs.^20^

Among the above‐mentioned studies, core/shell structures between binary II‐VI nanocrystals (NCs) have been proved to be the most effective and practical way to control the stability and suppress the nonradiative Auger recombination of II‐VI semiconductor QDs at the same time.^21^ To this end, core/shell structures have been widely explored between binary II‐VI nanocrystals, such as CdSe/CdS,^22^ CdSe/ZnS,^23^ and PbS/CdS.^24^ The shell can protect the core materials from harsh environment, so that the stability will increase. In general, there are three different types of Auger recombination: intradot Auger, trap‐assisted Auger, and diffusion‐assisted Auger.^25^ In case at least two excitons are generated within a single quantum dot, the intradot biexciton Auger recombination can occur via nonradiative transfer of the energy generated by electron and hole recombination in one exciton to the extra carrier, and the transferred energy will, in turn, be lost rapidly via thermal relaxation of the hot charge carrier. In trap‐assisted Auger recombination, one charge carrier in an exciton is localized by a trap, and energy is subsequently transferred to another charge carrier to eject from the QD. In diffusion‐assisted Auger recombination, the nonradiative Auger recombination is caused by the extra charge carriers diffusing from other QDs.^26^ Therefore, no matter which kind of Auger recombination, in order to block nonradiative Auger relaxation pathways, the ionization of QD should be prevented and the whole QD should keep neutral, i.e., the escape of photoexcited carriers from the QD must be prevented.^17^ Core/shell structure can forbid carrier's escape from the surface of QD through the wider bandgap provided by the shell material, so the valence bands (conduction bands) of shell are lower (or higher) than that of core. Thus, the Auger recombination can be suppressed essentially. However, this attempt has not yet succeeded in individual halide perovskite QDs.

Inspired by the successful achievement in II‐VI binary QDs, in this paper, aiming to enhance the structural stability and suppress nonradiative Auger recombination simultaneously, we synthesized novel core/shell structured colloidal perovskite QDs by covering CsPbBr_3_ QDs with II‐VI semiconductors. We carefully chose precursors and devised an effective methodology to fabricate perovskite semiconductor core/shell QDs. In particular, by capping CsPbBr_3_ QDs with CdS, we demonstrate greatly enhanced stability of CsPbBr_3_/CdS core/shell QDs even in the constant humidity of 75%. Remarkably, significant nonblinking characteristics have been achieved and narrow‐band emission with high PLQY has been maintained, indicating efficient reduction of nonradiative Auger recombination. For practical applications, we demonstrate amplified spontaneous emission (ASE) with enhanced efficiency under both one‐ and two‐photon excitation, together with stable, low‐threshold and high‐quality lasing. Therefore, successful fabrication of core/shell perovskite semiconductor QDs offers an exciting playground to develop optoelectronic devices for real applications.

CsPbBr_3_ QDs have been fabricated through the most common synthesis method developed by Protesecu et al.^3^ The obtained CsPbBr_3_ QDs with regular cubic shape were prepared as the core. Based on continuous injection method, we introduced a novel structure to overcome the blinking and instability drawbacks of CsPbBr_3_ QDs by growing thin CdS shell on the surface of CsPbBr_3_ core (see **Figure** **1**a). We introduced Cd‐oleate and sulfur as shell precursors and then successfully synthesized high‐quality CsPbBr_3_/CdS core/shell QDs. As the X‐ray diffraction (XRD) patterns (Figure 1b) clearly show, two types of crystalline structures were formed, with CsPbBr_3_ in a cubic structure (JCPDS #54‐0752) and CdS in zinc blende structure (JCPDS #89‐0440). Compared to the pure CsPbBr_3_ XRD pattern, extra peaks of CsPbBr_3_/CdS pattern match well with the standard XRD pattern of CdS, which is different from the CdSe/CdS (ZnS) core/shell. In case of CdSe/CdS core/shell, the XRD patterns of the CdSe and CdS (ZnS) are similar and the shell is thin, only resulting in the slight shift of XRD peaks.^27^, ^28^ However, in the case of CsPbBr_3_/CdS, their structures are totally different, and thus the peaks of the CdS could be observed. Similar results have been observed in the previously reported Fe_3_O_4_/Au (or Co/CdSe) core/shell structures.^29^, ^30^ Furthermore, we confirmed that CdS crystallizes in cubic zinc blende rather than hexagonal structure, as suggested by the location of (200) for cubic and lack of (100) and (101) for hexagonal. Figure S1 in the Supporting Information shows the appearance of a new lattice plane (111) in CsPbBr_3_/CdS QDs when compared with CsPbBr_3_ QDs.

**Figure 1:**
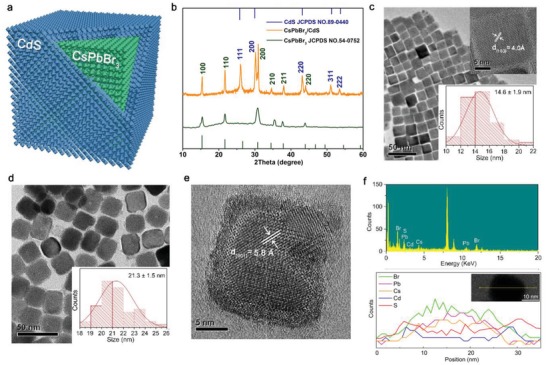
Morphology, composition, and crystal structure characterization of pure CsPbBr_3_ and obtained CsPbBr_3_/CdS. a) 3D schematic representation of core/shell structured CsPbBr_3_/CdS QDs. b) X‐ray diffraction pattern of CsPbBr_3_/CdS QDs (orange). The stick patterns show the standard peak positions of CsPbBr_3_ (bottom green sticks) and CdS (top blue sticks). c) TEM image of CsPbBr_3_, the insets show the HRTEM image and the particle size distribution statistics of CsPbBr_3_. d) TEM image of CsPbBr_3_/CdS QDs, the inset shows the particle size statistics of CsPbBr_3_/CdS QDs. e) HRTEM image of CsPbBr_3_/CdS QDs. f) EDX spectra of the CsPbBr_3_/CdS QDs.

To further verify the CsPbBr_3_/CdS core/shell structure, transmission electron microscope (TEM) and energy dispersive X‐ray (EDX) spectroscopy have been employed, with images shown in Figure 1c–f. The lattice distance of CsPbBr_3_ QDs was 4.0 Å, which corresponds to the (110) lattice spacing of the cubic phase. Started from an average size of 14.6 nm for CsPbBr_3_ cores (Figure 1c), these QDs were capped with the CdS shells individually via a modified literature procedure^29^ and the as‐prepared CsPbBr_3_/CdS core/shell QDs have an average size of 21.3 nm in a cubic shape. Furthermore, the size of the CsPbBr_3_ crystalline domains from the shown XRD patterns was evaluated by using the Scherrer formula (*D* = *Kλ*/(B cosθ)), which is 13.7 nm, consistent with size estimated by TEM. From the TEM images, it could be seen that the synthesized CsPbBr_3_/CdS QDs remained in cubic shape after they were dispersed in toluene for a week. Point angle of the cubic shape become circular arc, which further suggests the formation of CdS shell (Figure 1d). High‐resolution TEM in Figure 1e clearly shows the uniform CdS thin layer with an average thickness of ≈2.5 nm, fully grown on the surface of the CsPbBr_3_ QDs with the lattice distance of 5.8 Å corresponding to the (100) lattice spacing of CsPbBr_3_. No distinct lattice pattern of CdS layer has been observed, which is probably due to the thinness of the layer and the background noise. Existence of Cs, Pb, Br, Cd, and S elements were studied by mapping analysis (Figure S2, Supporting Information), where Cd and S were uniformly distributed on the surface of CsPbBr_3_/CdS QDs. And some bright inclusions on the surface of the core/shell CsPbBr_3_/CdS NCs are Pb not CdS seeds, which can also be observed in the TEM images of the pure CsPbBr_3_ NCs, as shown in Figure 1c. Besides, similar phenomenon has also been observed in many previous works done by other groups.^31^, ^32^, ^33^ The surface compositions of the CsPbBr_3_/CdS QDs were characterized by X‐ray photoelectron spectroscopy (XPS) analysis. As shown in Figure S3 in the Supporting Information, the emergence of Cd‐3d and S‐2p peaks further validates the proposed core/shell structure in which CdS acts as the shell encapsulating the CsPbBr_3_ core QDs. EDX spectroscopy analysis (Figure 1f) confirms the existence of five elements of Cs, Pb, Br, Cd, and S. In particular, the line scan results of S and Cd for one typically single element show relatively high counts at the two ends of the shell, which further prove the structure of core/shell. Based on the above analysis, we think that the synthesized CsPbBr_3_/CdS QDs are the core/shell structure with the cationic slight interdiffusion at the interface between the CsPbBr_3_ core and the CdS shell.

In order to explore atomic electronic structures for the synthesized CsPbBr_3_/CdS core/shell QDs, we have performed first‐principles calculations based on density function theory (DFT) to reveal the detailed interfacial geometry associated with electronic properties. We constructed CsPbBr_3_/CdS interfaces by using CsPbBr_3_ (100) surfaces with both CsBr and PbBr_2_ terminations, with which CdS thin films in (100) direction truncated by Cd or S atoms are bonded. Different adsorption geometries have been considered systematically to find the ground‐state configurations with the lowest total energy, and then the associated electronic structures were calculated. **Figure** **2** shows the optimized four ground‐state interfaces for CsPbBr_3_/CdS core/shell QDs, together with their interfacial density of states (DOS). Clearly, strong chemical bonding with drastic structural changes around the contact region were observed for all interfaces. The calculated bonding distances for interface of S plane of CdS in contact with CsBr plane of CsPbBr_3_, Cd with CsBr, Cd with PbBr_2_, and S with PbBr_2_ are 2.28, 2.19, 1.78, and 3.16 Å, respectively. To investigate the structural stability, we calculated the binding energy between CdS and CsPbBr_3_, which is defined as, *E* = [*E*(CdS) + *E*(CsPbBr_3_) − *E*(CsPbBr_3_/CdS)]/*A*, where *E*(CdS), *E*(CsPbBr_3_), and *E*(CsPbBr_3_/CdS) are the total energies of CdS films, CsPbBr_3_ surfaces, and CsPbBr_3_/CdS interfaces, respectively, and *A* is the surface area. The calculated *E* equals to 60, 14, 17, and 59 eV Å^−2^ for the four interfaces, which suggest the possible existence of core/shell QDs, and bonding of S atoms of CdS with CsPbBr_3_ surface (no matter with CsBr or PbBr_2_ termination) is significantly stronger.

**Figure 2:**
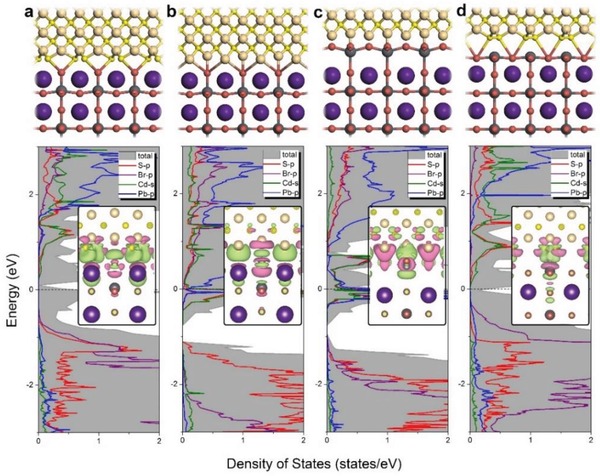
Four optimized local structures and partial DOS including charge density difference near the contact region. a) Up panel: optimized local structures of core/shell CsPbBr_3_/CdS QDs with S plane of CdS in contact with CsBr plane in CsPbBr_3_. Down panel: partial DOS projected onto CdS and CsPbBr_3_. In the inset, charge density difference near the contact region included (isovalue = 0.0008 e Å^−3^). Red color denotes charge accumulation and green denotes charge depletion. b–d) Interfaces for Cd in contact with CsBr, Cd with PbBr_2_, and S with PbBr_2_ plane, respectively.

The calculated DOS of the four interfaces for CsPbBr_3_/CdS QDs show strong overlap within both valence and conduction bands, suggesting strong chemical hybridization between two materials. By projecting total DOS onto the s orbital of Cs, p orbitals of Pb, Br, and s, and d orbitals of Cd, we found that the chemical bonding mainly involves p orbitals of S, Pb, Br elements and s orbitals of Cd, resulting in strong orbital hybridization. Figure 2 also includes the charge density difference plots, from which we can see significant charge redistribution around the bonding region. By careful examination of the symmetry and shape of charge density contour, we found the specific atomic orbitals for hybridization, in accordance with the partial DOS analysis.

Electrons/holes near the conduction/valence band edges are important for carrier transport and optical excitation. To visualize these states in real space, we calculated the charge densities near band edges of CsPbBr_3_/CdS interfaces (Figure S4, Supporting Information). Interestingly, we observed conduction and valance band edge states are spatially separated for all interfaces, which may have great influence toward the optical excitation and lifetime, for which the excited electrons could be effectively prevented from recombination due to the spatially different occupation of conduction/valence band edges. Figure S5 in the Supporting Information shows the plane‐averaged microscopic electrostatic potential near the interfacial region, where we see obvious potential change for all interfaces. Electrostatic potential drop or increase results in in‐built electric field, which may greatly facilitate separation of electron and hole pairs in CsPbBr_3_/CdS QDs. As we shall see later, these unique electronic structures will have great impact toward the physical properties of the synthesized QDs. In addition, the separated conduction/valence band edges lead to a separation of electrons and holes, but the wider bandgap of CdS limits their actions, the photoexcited carriers are hard to escape to the surface of QDs, and the carriers can only combine by radiative recombination. Therefore, the QDs should always be neutral and the incentive of auger nonradiative recombination was gone, and the nonblinking QDs can be obtained theoretically.

Next, we turn to investigate the optical properties of the core/shell structured CsPbBr_3_/CdS. The bandgap of cubic phase CdS QDs is ≈2.62 eV,^34^ which is wider than that of CsPbBr_3_ QDs (≈2.4 eV).^35^ The valence bands (conduction bands) of shell are lower (or higher) than that of core and can effectively improve the stability of quantum dots in harsh environments if high quality and epitaxy growth between the shells and single‐crystalline core nanocrystals can be achieved.^36^ To prepare for the sample, the as‐grown CsPbBr_3_/CdS QDs were dispersed in toluene and deposited on glass sheet. As shown in **Figure** **3**a, after being capped with CdS, both PL and absorption spectra are blueshifted as compared to those of pure CsPbBr_3_ QDs, suggesting that CdS not only acts as one distinct and protective shell, but also leads to some etching to the emitting core (Figure S6, Supporting Information). It can be attributed to the diffusion of cadmium ions into the core material and the partial substitution of cadmium ions for plumbum ions, resulting in an increase of the bandgap energy. However, the degree of etching is slight. Similar results have been shown in other core/shell structured work.^29^, ^30^ The full‐width half‐maximum (FWHM) of CsPbBr_3_/CdS QDs was measured to be 20 nm, similar to those of pure CsPbBr_3_ QDs. CsPbBr_3_ QDs are able to show narrow FWHM and high PLQY at the same time while such coexistence of these two features can only be seen in core/shell structured QDs in the past, like CdSe/CdS.^3^ The PLQY of pure CsPbBr_3_ QDs was measured to be 90%, while CsPbBr_3_/CdS QDs have only a slightly decrease to 88%. Narrow FWHM of QDs improves the spectral resolution of QD‐based illumination or display, and it also increases the number of wavelength detection channels for multiplexing applications. Time‐resolved PL decays of QDs (Figure S7, Supporting Information) demonstrate an average decay time of 17.6 ns for CsPbBr_3_ and 22.8 ns for CsPbBr_3_/CdS QDs. The result shows that the CsPbBr_3_ QDs exhibit faster decay time (considering two decay times of 7.5 and 35 ns) than CsPbBr_3_/CdS QDs (considering two decay times of 10.4 and 26.7 ns). The longer time and the shorter time are attributed to radiative recombination and recombination through traps or surface states, respectively.^37^ According to the ratio of nonradiative recombination between two obtained materials (97% for CsPbBr_3_ QDs and 91% for CsPbBr_3_/CdS QDs), our synthesized CsPbBr_3_/CdS QDs had a relatively low nonradiative recombination, suggesting lower Auger recombination and fluorescence intermittency.

**Figure 3:**
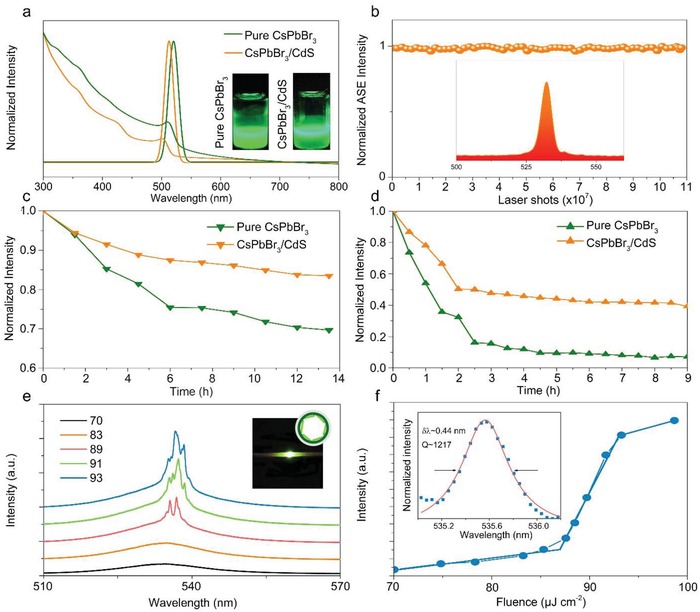
Optical properties and photoluminescence stability test of pure CsPbBr_3_ and obtained CsPbBr_3_/CdS. a) Photoluminescence and optical absorption spectra of pure CsPbBr_3_ (green curve) and CsPbBr_3_/CdS (orange curve) in toluene. λ_ex_ = 350 nm, λ_em_ = 519 nm (CsPbBr_3_ QDs), λ_em_ = 513 nm (CsPbBr_3_/CdS QDs), the insets show the photograph (λ_ex_ = 360 nm) of luminescent CsPbBr_3_ (left) and CsPbBr_3_/CdS (right) respectively. b) Plot of emission intensity under continuous pulsed laser irradiation up to 3 h (up to 1.08 × 10^7^ excitation cycles). The inset displays the ASE spectrum of the CsPbBr_3_/CdS excited by 800 nm fs laser with pumping intensity of 980 µJ cm^−2^. c) PL intensity change under 75% constant humidity for 13.5 h. d) PL intensity change under 60 °C for 9 h. e) Intensity‐dependent emission spectra from CsPbBr_3_/CdS around the lasing threshold. Inset: the photograph of CsPbBr_3_/CdS QDs incorporated in a cylindrical microcapillary above lasing threshold and the WG modes supported by the microring resonator. f) The integrated emission intensity plotted as a function of excitation intensity. Inset: Lorentz fitting (solid red line) of a lasing oscillation mode (blue square).

To explore the stability of CsPbBr_3_/CdS QDs, we conducted experiments under constant intense laser irradiation, high humidity, and high temperature for several hours under atmospheric condition. The emission intensity variation of CsPbBr_3_/CdS QDs under continuous excitation of 800 nm fs laser pulses (pumping intensity is 0.98 mJ cm^−2^) for at least 3 h (up to 1.08 × 10^7^ excitation cycles) is shown in Figure 3b. Surprisingly, the ASE intensity of CsPbBr_3_/CdS QDs shows extremely small fluctuation and keeps stable. To test stability under high humidity, we kept the pure CsPbBr_3_ QD and CsPbBr_3_/CdS QD films being stored under dark field, 75% RH environment at room temperature to record the PL intensity every 90 min (up to 13.5 h) (Figure 3c). The PL intensity of pure CsPbBr_3_ QDs quenches much faster than CsPbBr_3_/CdS QDs, with pure CsPbBr_3_ decaying to 65% while CsPbBr_3_/CdS QDs still kept 83% after 14 h. Thermal stability of CsPbBr_3_/CdS QDs was measured under a constant temperature at 60 °C, and the intensity variations were recorded for every 30 min. We found that CsPbBr_3_/CdS QDs always exhibited much better stability than pure CsPbBr_3_ QDs. When the time trace comes to 9 h, the PL intensity of pure CsPbBr_3_ QDs becomes nearly zero, while CsPbBr_3_/CdS QDs remain about 40% (Figure 3d). As shown in Figure S8 in the Supporting Information, the emission peaks stay the same with the fresh prepared CsPbBr_3_/CdS QDs after 3 months. Additionally, the water‐resistant ability of QDs was also checked, which was done by gradually adding deionized water into samples with toluene as solvent. It was observed that PL intensity of pure CsPbBr_3_ QDs decreased immediately compared to that of CsPbBr_3_/CdS QDs, and finally got quenched. However, CsPbBr_3_/CdS QDs showed strong PL when contacting with water, exhibiting much better stability than the pure CsPbBr_3_ QDs (Figure S9, Supporting Information). These results confirmed that capping CsPbBr_3_ QDs with CdS can significantly enhance the stability of perovskite in both high temperature and humidity stability, making CsPbBr_3_/CdS QDs an excellent candidate for practical optoelectronic applications in future.

Variation in fluorescence intensity with time (fluorescence intermittency, or “blinking”) is one of the key limiting factors for QDs.^38^ As previously reported,^39^ individual CsPbBr_3_ QD has relatively long low‐emissivity OFF states, which may impede the application of QDs in biological settings for tracking single‐photon sources and LEDs. Here, to demonstrate the blinking phenomenon, a dilute solution of the CsPbBr_3_ QDs was mixed with a 2% chloroform solution of polymethyl methacrylate (PMMA) and spin‐coated onto a coverslip, and then the CsPbBr_3_ QDs were excited under constant irradiation of 445 nm laser in air. As shown in **Figure** **4**a and Video S1 in the Supporting Information, pure CsPbBr_3_ QDs spend most of their time in low‐emissivity OFF state and show only short bursts toward high‐emissivity ON state. The average OFF time fraction is nearly 37%. The ON and OFF states have formed blinking phenomenon of QDs, and the “blinking” can be attributed to the fluctuations in net charge inside or around the QDs.^38^, ^39^ The same preparation steps were used for CsPbBr_3_/CdS QDs. In contrast, blinking behaviors of CsPbBr_3_/CdS QDs are substantially improved. As shown in Figure 4e and Video S2 in the Supporting Information, CsPbBr_3_/CdS QDs exhibit a distinct nonblinking feature and keep ON state nearly 450 s with no twinkle state. The average ON time fraction is larger than 99% (inset in Figure 4b) and nearly no “gray states” (which have been attributed to the emission from positive trions^40^) could be observed. It has been reported that the trap‐assisted Auger recombination exhibited faster recombination rates than those exhibited by a typical biexciton; hence, the trap‐assisted Auger is particularly detrimental to the photoluminescence process and hence the PLQY.^41^ Therefore, the results in the present study reveal that deep electron or hole traps have been significantly reduced with the core/shell structure, consistent with the high ON time fraction measured for exciton emission.^22^ The second‐order photon correlation experiments for the CsPbBr_3_/CdS QDs were performed by using a time‐correlated single‐photon counting system. As shown in Figure 4c, the *g* ^2^(*t*), the value of normalized second‐order correlation function, being 0.43 is smaller than 0.5, confirming the single‐dot measurements using the continuous wave laser were within single‐exciton regime.^42^ Figure 4d,e shows three screenshots cut from the fluorescent video to illustrate the fluorescent images of pure CsPbBr_3_ and CsPbBr_3_/CdS QDs, respectively, which clearly show the variation in PL intensity of single dot.

**Figure 4:**
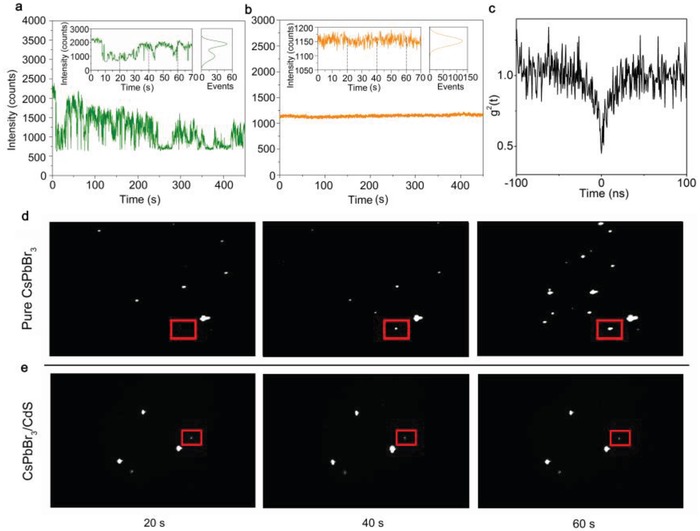
Blinking behavior of pure CsPbBr_3_ and obtained CsPbBr_3_/CdS. a,b) PL intensity time trace of pure CsPbBr_3_ (green curve) and CsPbBr_3_/CdS (orange curve). The insets in (a) and (b) show the PL intensity time trace in 70 s and PL intensity distribution of pure CsPbBr_3_ (green curve) and CsPbBr_3_/CdS (orange curve), respectively, indicating the ON and OFF states. c) Antibunching curves of the single CsPbBr_3_/CdS QDs in (b). The data were recorded by a single‐photon counting system with two avalanche photodiodes. d,e) Optical photograph of pure CsPbBr_3_ and CsPbBr_3_/CdS under constant irradiation of 445 nm laser in air, the exposure time is 100 ms. The single dot in the red box stands for the PL intensity time trace data we chose for (a) and (b).

Different from the photoluminescence process, ASE is a kind of stimulated emission process directly related to lasing performance. In disordered gain systems, ASE resulting from the amplification of spontaneously emitted photons by stimulated emission harnesses optical gain provided via light scattering that is induced by intrinsic disorder in the medium before light output.^43^ Its performance characteristics such as the threshold and emission efficiency are important figure‐of‐merit indicating the light amplification ability in the material and the optimized quality of lasing performance. In addition, recently, low‐threshold frequency upconverted emission by two‐photon excitation in nanostructures has attracted increasing scientific interest, because it provides a novel frequency upconversion approach and weeds out the phase‐matching condition.^44^ Due to the excellent optical properties, all‐inorganic CsPbX_3_ is expected to be an excellent candidate for two‐photon‐excited upconversion devices. In the present study, the ASE performance was investigated under one‐ and two‐photon excitation at room temperature using CsPbBr_3_/CdS QD and pure CsPbBr_3_ QD samples deposited on glass, respectively. In order to avoid the complication of reabsorption and amplification effects, the excitation energy was kept low. Thresholds (*P*_th_) of two‐photon pumped ASE from the CsPbBr_3_/CdS QDs and pure CsPbBr_3_ QDs are determined to be about 700 and 730 µJ cm^−2^. Under relatively low pump excitation (lower than threshold), broad spontaneous emission peak centered at ≈514 nm with an FWHM of 20 nm can be obtained for CsPbBr_3_/CdS QDs. As long as the pump intensity is above ASE threshold, a relatively redshift sharp peak appears at ≈531 nm for CsPbBr_3_/CdS QDs, and FWHM decreases from 20 to ≈4 nm (Figure S10, Supporting Information). Similar ASE results can be obtained under the one‐photon excitation, as shown in Figure S11 in the Supporting Information. As compared with the pure CsPbBr_3_ QDs, both samples exhibit similar emission features under both one‐ and two‐photon excitation. Specifically, the emission intensity increases dramatically and the FWHM decreases sharply (both from 20 to 4 nm for two‐photon excitation and from ≈28 to ≈6 nm for one‐photon excitation) when the pumping intensity is increased above the *P*_th_ (Figures S10 and S11, Supporting Information), except that the CsPbBr_3_/CdS QDs have lower thresholds both under one‐ and two‐photon excitation. As we compare the output intensity (*P*_out_) in detail, we notice that the *P*_out_ of CsPbBr_3_ at 1.5P_th_ (1.095 mJ cm^−2^) is 1.6 times of *P*_out_ at its *P*_th_, while the *P*_out_ of CsPbBr_3_/CdS at 1.5P_th_ (1.05 mJ cm^−2^) is 3.7 times of the *P*_out_ at its *P*_th_. Therefore, after being capped with semiconductor shell, the ASE threshold of CsPbBr_3_ has decreased to 30 µJ cm^−2^ and relative efficiency increased by 131%.

In general, for the potential application in stimulated emission, such as ASE and lasing, the characteristic of optical gain from laser medium is extremely important. Unfortunately, the optical gain of QDs are always limited by the nonradiative biexciton (multicarrier) Auger recombination,^45^ which is especially severe as the excitation intensity becomes higher. If extra carriers exist, the energy released during electron–hole recombination could be nonradiatively transferred to it, rather than contribute to the generation of photon. It also has been revealed that the intradot Auger recombination can be suppressed only by means of band engineering to manipulate wave functions that reduce the matrix element representing the Auger recombination process^41^ in Auger lifetime. Therefore, the lower *P*_th_ and higher ASE efficiency of CsPbBr_3_/CdS obtained in the present study indicate the competition between the stimulated emission and the nonradiative Auger recombination becomes mitigated, due to the suppressed intradot biexciton Auger recombination in the core/shell structure. These results indicate that CsPbBr_3_/CdS QDs hold great potential for high‐quality lasers and optoelectronic devices under both one‐ and two‐photon excitation.

In order to further demonstrate the great potential for high‐quality lasers, we incorporated CsPbBr_3_/CdS QDs into the cylindrical microcapillary (inset of Figure 3e). When pump intensity is below 87 µJ cm^−2^, the broadband emission from CsPbBr_3_/CdS QDs is centered at ≈514 nm with the FWHM of 20 nm. Along with the increase of pump fluence, the emission spectrum increases. When pump intensity exceeds 87 µJ cm^−2^, a series of sharp peaks appear at around 536 nm of the emission spectrum and the intensities of these peaks increase sharply along with the increase of excitation intensity (Figure 3e). Specifically, the spontaneous emission first appeared in the PL spectrum when pump intensity was below 87 µJ cm^−2^. As long as the excitation is increasing, ASE is achieved at first. When the pump intensity increases further, stable lasing action takes place. The typical “S”‐curve shape^46^ is obtained as expected when the pump fluence dependence of the integrated emission intensity is plotted in log scale (Figure 3f), which reveals the three‐step evolution from spontaneous emission via amplified spontaneous emission to stable laser oscillation in microcavity resonators coupled with CsPbBr_3_/CdS QDs. In order to obtain the quality (*Q*) factor, an individual lasing peak is selected and analyzed. The individual peak at 535 nm shows an FWHM ≈0.44 nm, and then the Q factor is determined to be 1217 by simple calculation of *Q* = λ/λ_FWHM_ (inset in Figure 3f), where λ is the peak wavelength and λ_FWHM_ is the peak width. The high *Q* factor and evenly distributed lasing peaks are likely related to the whispering gallery modes arising from the total internal reflections at the interfaces between the inner tubular surfaces and CsPbBr_3_/CdS QDs.^47^

In summary, we have successfully synthesized colloidal perovskite QDs with a unique core/shell structure by capping individual CsPbBr_3_ QD with CdS shell. We show that these CsPbBr_3_/CdS core/shell QDs possess striking advantages of high uniformity, improved operational stability, nonblinking characteristics, narrow‐band emission, and high PLQYs simultaneously. Theoretical calculations based on DFT reveal the atomic interface structure and support the existence of CsPbBr_3_/CdS core/shell structure. ASE of CsPbBr_3_/CdS QDs exhibits a relatively lower threshold under both one‐ and two‐photon excitation, and the relative efficiency increases by over 130% with respect to that of pure CsPbBr_3_ QDs. Both the nonblinking PL and improved ASE provide convincing evidence that the nonradiative Auger recombination has been suppressed. Furthermore, stable low‐threshold whispering‐gallery‐mode lasing with high‐quality factor has been achieved by incorporating CsPbBr_3_/CdS QDs into microtubule resonators. All these improvements indicate that our strategy of core/shell colloidal perovskite QDs may provide a versatile, stable platform for the generation of nonblinking perovskite materials without sacrificing their efficient optical performance, and hence for future exploration and development of LEDs, low‐threshold lasers, and solar cells.

## Experimental Section

*Materials*: Cs_2_CO_3_ (Aldrich, 99.9%), PbBr_3_ (ABCR, 98%), cadmium (II) oxide (Sigma Aldrich, 99.5%), oleic acid (Sigma Aldrich, 90%), 1‐octadecene (ODE, Sigma Aldrich, tech. 90%), sulfur (Sigma Aldrich, 99.98%), oleylamine (OLAM, Sigma Aldrich, tech. 70%), and toluene (Sigma Aldrich, 99.8%) were acquired.

*Cd‐Oleate Solution Synthesis*: For the synthesis of Cd‐oleate solution, a protocol by Li et al.^29^ was followed. A 0.38 m Cd‐oleate solution was made by dissolving 383 mg CdO in 3.9 mL OA and 3.9 mL ODE at 280 °C under N_2_ flow. After 1 h, the CdO was dissolved and the clear solution was degassed for 30 min at 110 °C.

*CsPbBr_3_/CdS Core/Shell QDs*: For the synthesis of CsPbBr_3_ core QDs, a protocol by Protesescu et al.^3^ was followed. 100 mg Cs_2_CO_3_ was loaded into 100 mL three‐neck flask along with 4 mL ODE, and 0.5 mL OA, dried for 1 h at 120 °C, and then heated under N_2_ to 150 °C until all Cs_2_CO_3_ reacted with OA. Since Cs‐oleate precipitated out of ODE at room temperature, it had to be preheated to 100 °C before injection. 5 mL ODE and 69 mg PbBr_2_ were loaded into 100 mL three‐neck flask and dried under vacuum for 1 h at 120 °C. 0.5 mL dried OLAM and 0.5 mL dried OA were injected at 120 °C under N_2_. After complete solubilization of a PbBr_2_ salt, the temperature was raised to 150 °C and Cs‐oleate solution (0.4 mL, 0.125 m in ODE, prepared as described above) was quickly injected and, 5 s later, the reaction finished. 3.6 mL ODE, 1 mL Cd‐oleate solution, and 0.4 mL 1 m sulfur in OLAM solution were mixed when the reaction for CsPbBr_3_ QDs finished, and added dropwise over 20 min to the CsPbBr_3_ solution at 150 °C under N_2_ flow. After the addition was complete, the mixture was allowed to react for 20 min at 150 °C, and was subsequently cooled down by ice‐water bath, washed with toluene, and dispersed in toluene.

*First‐Principles Calculations*: The first‐principles calculations were performed by using the projector augmented wave formalism of DFT method as implemented in the Vienna ab initio simulation package (VASP).^48^ A 400 eV cutoff for the plane‐wave basis set was adopted in all computations. Electronic exchange and correlation effects were treated by using the generalized gradient approximation (GGA) in Perdew–Burke–Ernzerhof (PBE) format.^49^ CsPbBr_3_/CdS core/shell QDs were simulated by a slab model consisting of 9/10‐layered CsPbBr3 surface and 13/14‐layered CdS thin films sitting on top of the surface. CsPbBr_3_ (001) surfaces in contact with CdS (001) were systematically studied with different contact geometries. During structural optimization, all the atoms were fully relaxed until the atomic forces were smaller than 0.01 eV Å^−1^. The Brillouin zone was represented by Gamma‐centered unit cells with 6 × 6 × 1 Monkhorst‐Pack k‐point mesh for geometry optimization of QD models.

*Optical Characterization*: Photoluminescence spectra were measured by Agilent Cary Eclipse spectrograph FLS920P and UV–vis absorption spectra were characterized by Shimadzu 2100 UV–vis spectrophotometer. XRD characterization was done by Shimadzu/6100 X‐ray diffractometer, using a Cu Kα radiation source (wavelength at 1.5405 Å). Transmission electron microscopic, high‐resolution transmission electron microscopic (HRTEM), and the elemental mapping images were taken on a JEOL‐2010 microscope with an accelerating voltage of 200 kV and an energy dispersive detector. Photoluminescence quantum yield was measured by Edinburgh fluorescence spectrometer FS5. Time‐resolved PL measurements were collected by fluorescence lifetime measurement system (QM/TM/NIR, PTI, America).

*PL Intensity Trace Measurement for Single QD*: All samples were studied under ambient conditions by a home‐built wide‐field fluorescence microscope. The 450‐nm laser line from an Ar+ CW laser was used for excitation. The excitation power was adjusted by a set of neutral density filters. The excitation area was ≈25 µm in diameter, covering many nanocrystals as shown in the photoluminescence image (Figure S1, Supporting Information). The PL was collected by an oil immersion objective lens (Olympus UPlanFLN 60×, NA = 1.25) and imaged on a charge coupled deviced (CCD) camera (Pro‐EM: 512B, Princeton Instruments) after passing through a long‐pass filter (HQ675lp, Chroma Technology Corp.). A transmission grating (150 S4 lines mm^−1^) was placed in front of the CCD camera for the spectral measurement giving a resolution of ≈8 nm. The PL intensity transients contained 1000 frames with an exposure time of 100 ms per frame.

*ASE and Lasing Experiment*: For the two‐photon‐pumping ASE experiments, a commercially supplied Ti:sapphire regenerative amplifier (800 nm, 35 fs, 1 kHz) was employed as the pumping source. For one‐photon excitation, a UV laser pulse centered at 400 nm was generated by frequency doubling in a 200 µm thick beta barium borate (BBO, type I, θ = 29.2°) crystal. In both experiments, the laser beams were focused onto the samples using a cylindrical lens with a focal length of 10 cm. The stripe length of the spot size could be precisely controlled by using an adjustable slit. The ASE was detected by a fiber spectrometer (Ocean Optics) with a spectral resolution of 1 nm. The lasing signals were detected using an Acton Spectra Pro SP‐2358 with a resolution of 0.14 nm.

## Conflict of Interest

The authors declare no conflict of interest.

## Supporting information

Supplementary

Supplementary

Supplementary
