# Supplementary material for: Single Halide Perovskite/Semiconductor Core/Shell Quantum Dots with Ultrastability and Nonblinking Properties
Source: Adv Sci (Weinh). 2019 Jul 1;6(18):1900412. doi: 10.1002/advs.201900412 (PMC6755528; doi:10.1002/advs.201900412)
Supplement: Supplementary file 1 — Supplementary [file ADVS-6-1900412-s002.pdf]

Copyright WILEY-VCH Verlag GmbH & Co. KGaA, 69469 Weinheim, Germany, 2019.

Supporting Information

for *Adv. Sci.,* DOI: 10.1002/advs.201900412

Single Halide Perovskite/Semiconductor Core/Shell Quantum Dots with Ultrastability and Nonblinking Properties

Xiaosheng Tang, Jie Yang, Shiqi Li, Zhengzheng Liu, Zhiping Hu, Jiongyue Hao, Juan Du, Yuxin Leng,* Haiyan Qin, Xing Lin, Yue Lin, Yuxi Tian, Miao Zhou,* and Qihua Xiong*

Copyright WILEY-VCH Verlag GmbH & Co. KGaA, 69469 Weinheim, Germany, 2019.

Supporting Information

Title

Single Halide Perovskite/Semiconductor Core/shell Quantum Dots with Ultrastability and Nonblinking Properties

Xiaosheng Tang, Jie Yang, Shiqi Li, Zhengzheng Liu, Zhiping Hu, Jiongyue Hao, Juan Du, Yuxin Leng*, Yue Lin, Yuxi Tian, Miao Zhou*, Qihua Xiong*

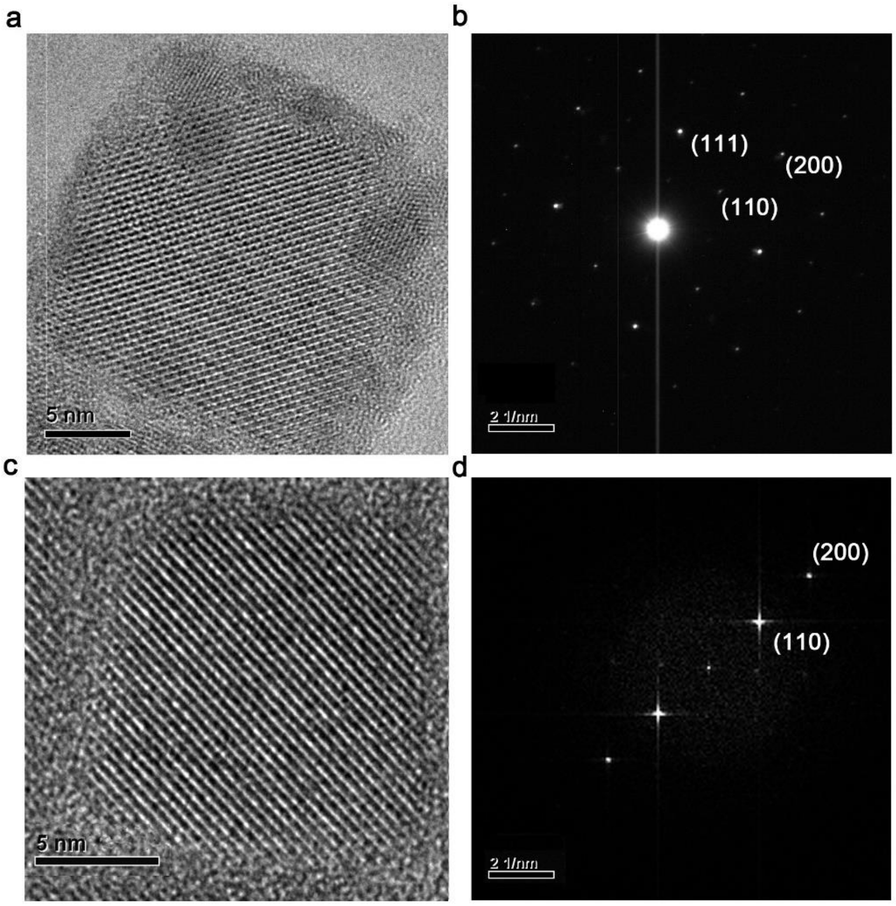

Figure S1. a) HRTEM image of $CsPbBr_3$/CdS. b) FFT patterns of the $CsPbBr_3$/CdS in a). c), HRTEM image of Pure $CsPbBr_3$. d) FFT patterns of the Pure $CsPbBr_3$ in c).

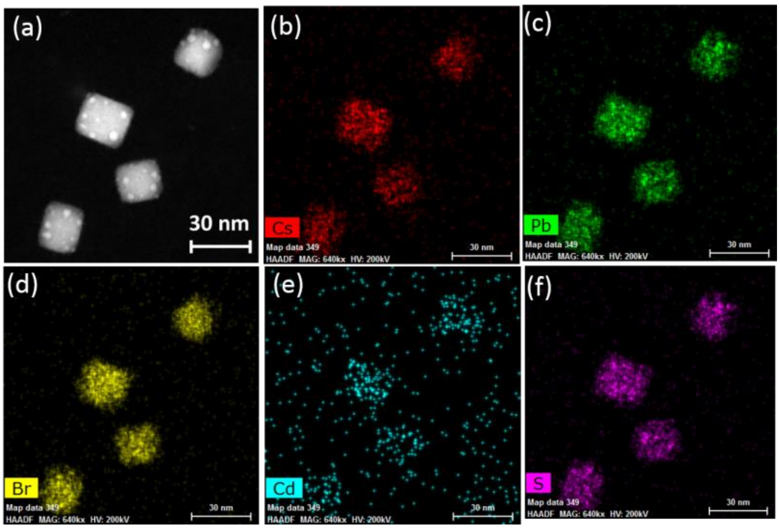

Figure S2. Mapping images of Cs, Pb, Br, Cd and S elements.

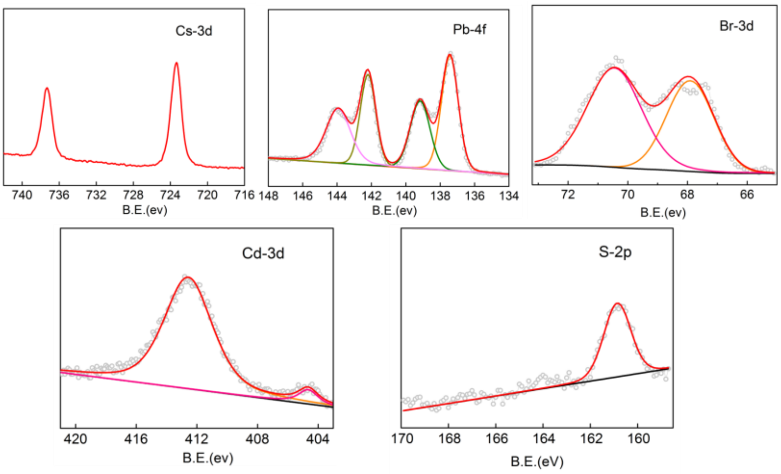

Figure S3 X-ray photoelectron spectra (XPS) of $CsPbBr_3$/CdS core/shell QDs

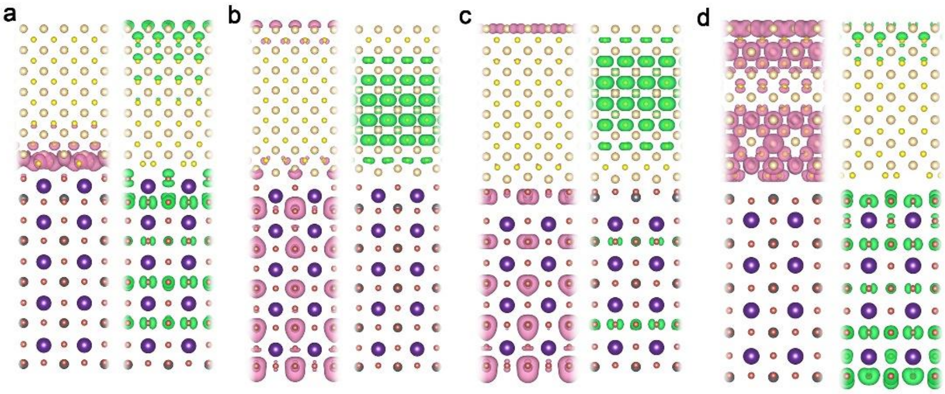

Figure S4. a) partial charge density of optimized local structure with S plane of Cd in contact with CsBr plane in $CsPbBr_3$. Left panel is conduction band edge and right panel is valence band edge. b-d) are Cd in contact with CsBr, Cd with $PbBr_2$ and S with $PbBr_2$ plane, respectively.

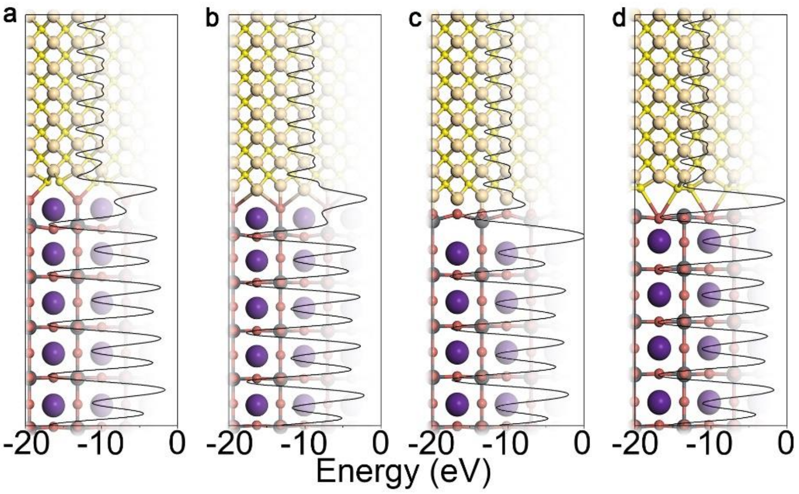

Figure S5. a-d) are electrostatic average potential of S in contact with CsBr, Cd in contact with CsBr, Cd with $PbBr_2$ and S with $PbBr_2$ plane, respectively.

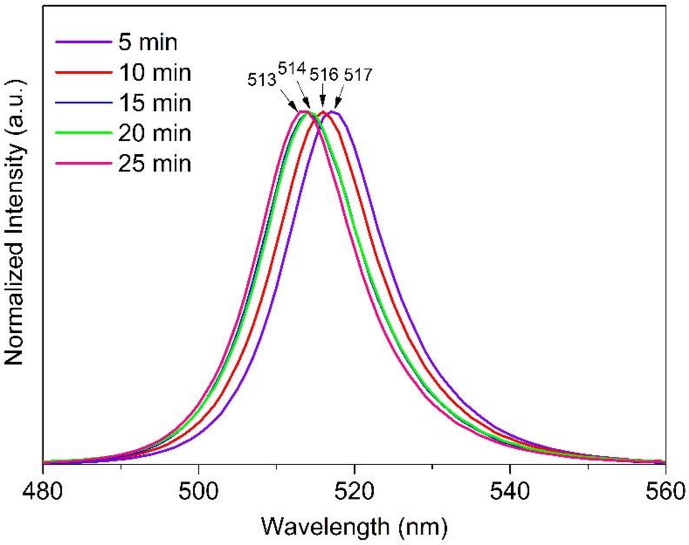

Figure S6. Evolution of emission spectra during a typical synthesis. The numbers correspond to reaction time in minutes. The arrows point to the corresponding peaks and indicate the peak values.

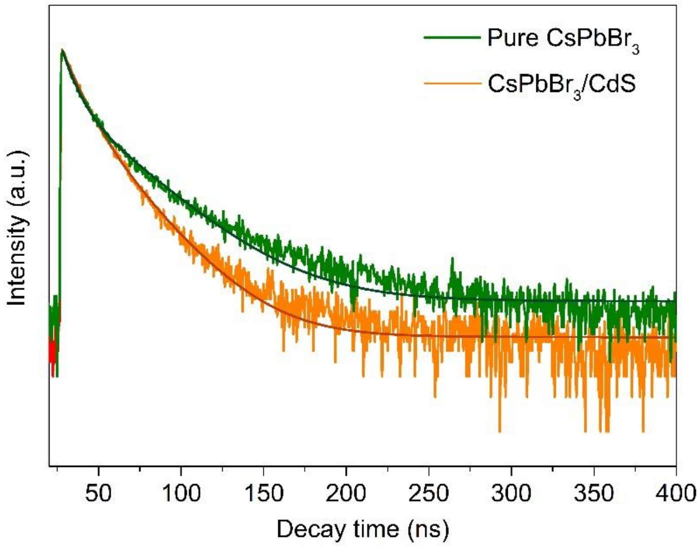

Figure S7. Time-resolved PL spectrum of $CsPbBr_3$ (green curve) and $CsPbBr_3$/CdS (orange curve) in toluene. $\tau_{CsPbBr3}$=17.6ns, $\tau_{CsPbBr3/CdS}$ =22.8ns. The ratio of non-radiative recombination is 97% for pure $CsPbBr_3$ and 91% for $CsPbBr_3$/CdS.

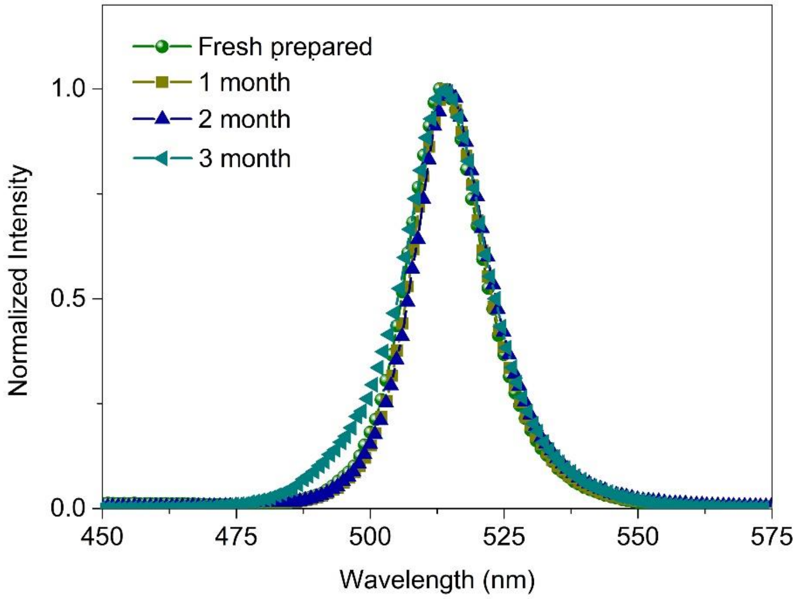

Figure S8. The variation of fluorescence spectrum of $CsPbBr_3$ /CdS QDs within 3 months.

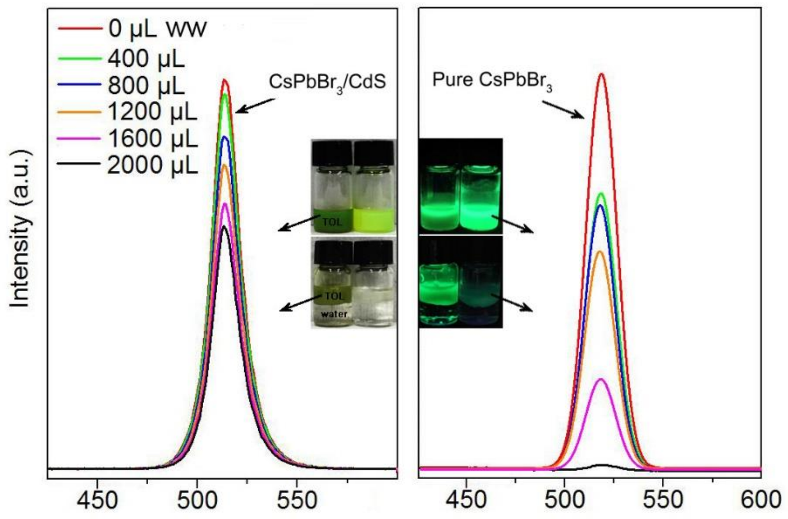

Figure S9. The variation of PL intensity with water (ww) added over and over (400 µL each time) of $CsPbBr_3$ QDs (right) and $CsPbBr_3$ /CdS (left). The insets show the photographs and PL photographs of $CsPbBr_3$ (right) and $CsPbBr_3$/CdS (left). The QDs dispersed in toluene (TOL).

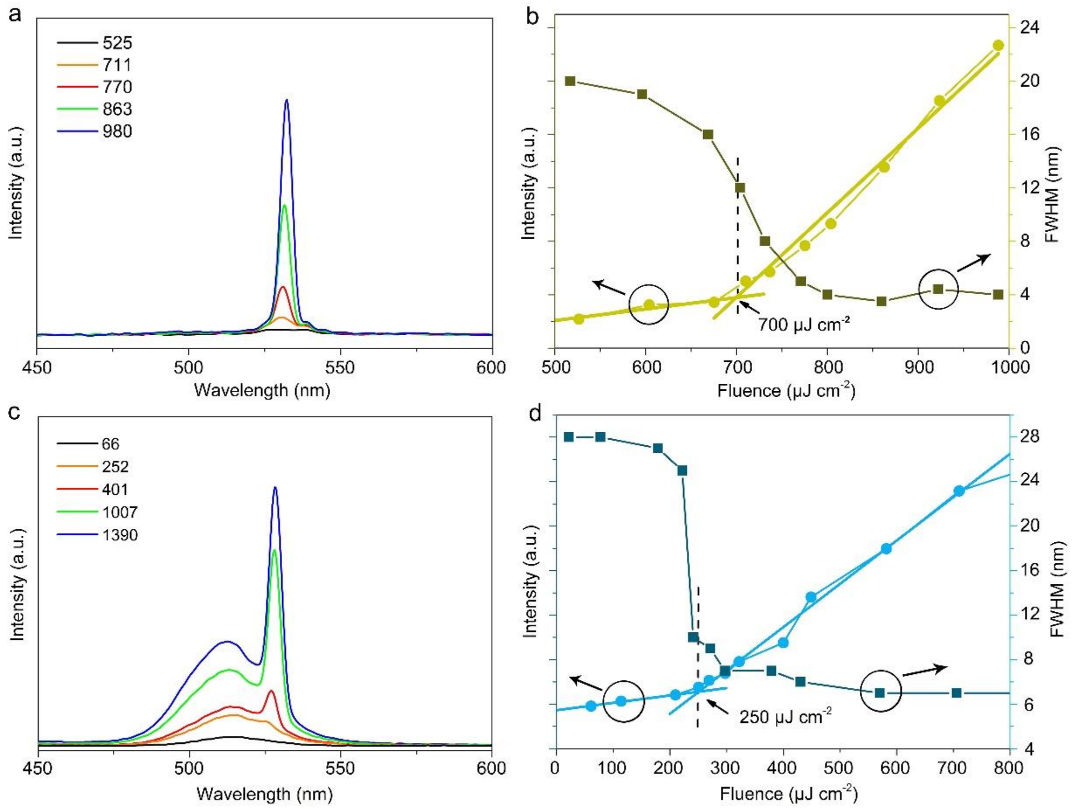

Figure S10. a) Pump-intensity dependence of the emission from the $CsPbBr_3$/CdS QDs under 800 nm fs laser excitation. b) Dependence of output intensity and linewidth as a function of pumping density under 800 nm fs laser excitation, showing the threshold of ~700 µJ cm^{-2}. c) Pump-intensity dependence of the emission from the $CsPbBr_3$/CdS QDs under 400 nm fs laser excitation. d) Dependence of output intensity and linewidth as a function of pumping density under 400 nm fs laser excitation, showing the threshold of ~250 µJ cm^{-2}.

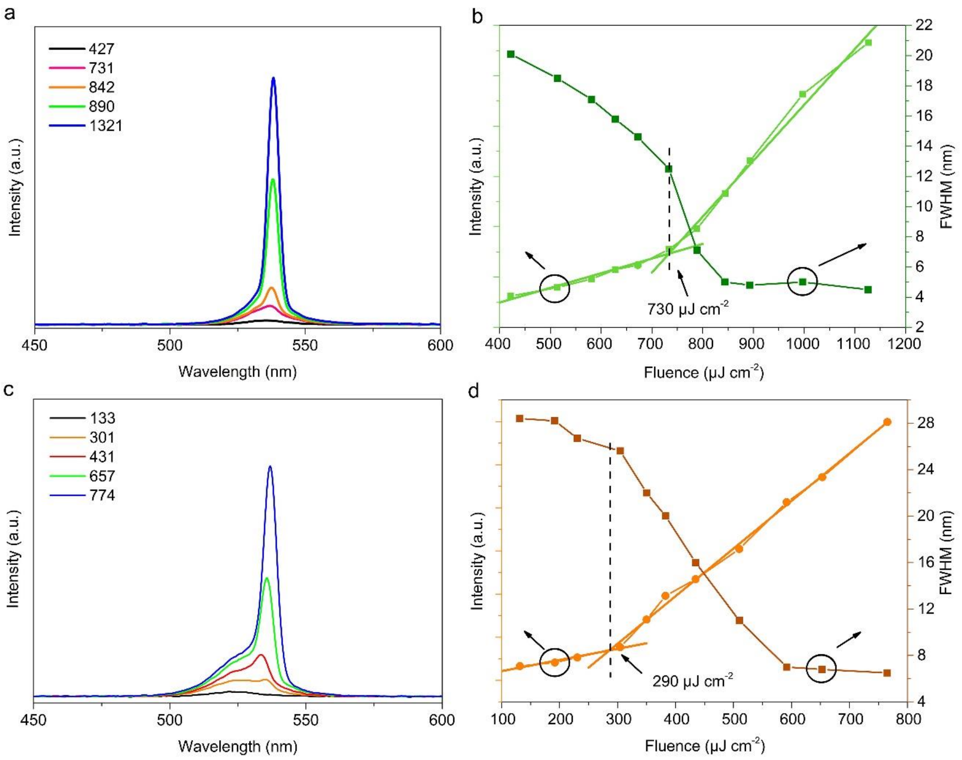

Figure S11. a) Pump-intensity dependence of the emission from the $CsPbBr_3$ QDs under 800 nm fs laser excitation. b) Dependence of output intensity and linewidth as a function of pumping density under 800 nm fs laser excitation, showing the threshold of ~730 μJ cm^{-2}. c) Pump-intensity dependence of the emission from the $CsPbBr_3$ QDs under 400 nm fs laser excitation. d) Dependence of output intensity and linewidth as a function of pumping density under 400 nm fs laser excitation, showing the threshold of ~290 μJ cm^{-2}.
